# Effects of Assistive Robot Behavior on Impressions of Patient Psychological Attributes: Vignette-Based Human-Robot Interaction Study

**DOI:** 10.2196/13729

**Published:** 2019-05-19

**Authors:** Meia Chita-Tegmark, Janet M Ackerman, Matthias Scheutz

**Affiliations:** 1 Tufts University Medford, MA United States

**Keywords:** robotics, emotional intelligence, patient-centered care

## Abstract

**Background:**

As robots are increasingly designed for health management applications, it is critical to not only consider the effects robots will have on patients but also consider a patient’s wider social network, including the patient’s caregivers and health care providers, among others.

**Objective:**

In this paper we investigated how people evaluate robots that provide care and how they form impressions of the patient the robot cares for, based on how the robot represents the patient.

**Methods:**

We have used a vignette-based study, showing participants hypothetical scenarios describing behaviors of assistive robots (patient-centered or task-centered) and measured their influence on people’s evaluations of the robot itself (emotional intelligence [EI], trustworthiness, and acceptability) as well as people’s perceptions of the patient for whom the robot provides care.

**Results:**

We found that for scenarios describing a robot that acts in a patient-centered manner, the robot will not only be perceived as having higher EI (*P*=.003) but will also cause people to form more positive impressions of the patient that the robot cares for (*P*<.001). We replicated and expanded these results to other domains such as dieting, learning, and job training.

**Conclusions:**

These results imply that robots could be used to enhance human-human relationships in the health care context and beyond.

## Introduction

With new advances in the fields of robotics and artificial intelligence, interest has grown in the introduction of robots as social agents in health care practice, especially for the management of chronic illness or geriatric conditions [1]. Socially assistive robots are machines designed to provide assistance through social means rather than physical ones, using social interactions for monitoring, coaching, providing companionship, and supporting health-promoting activities [2]. Robots are envisioned to play roles such as monitoring and record-keeping of symptom progression [3], helping with pill sorting and medication schedules [4], guiding people through therapeutic tasks [5], providing companionship [6], acting as stress reducers and mood enhancers [7], or supporting social interactions [8,9]. Although considerable attention has been given to the study of patient-robot interactions, less research has focused on the triangulation of patient-robot-others relationships, where *others* can be doctors, therapists, caregivers, or simply members of the society that the patient might interact with. As we design social robots for health care, we need to understand the effects these robots can have not only on the patient but also on the abovementioned *others* and how the robot fits overall into the network and dynamics of the patient’s social relationships. This is important because social life and support has been consistently shown to be a crucial predictor of health outcomes [10].

In this paper, we take the first steps toward studying the potential indirect effects of assistive social robots on the relationship between patients and others. We look at this through the lens of patient-centered care as a desirable approach [11] and emotional intelligence (EI) as a desirable set of capabilities for the robot [12-14].

In the first step of the study we investigated how robots can influence others’ impressions of a patient’s psychological attributes, asking, for example, whether people think of a patient as being competent, honest, and self-disciplined rather than disruptive, hostile, and disorganized. These psychological attributes have been shown to make a difference in the quality of care a patient may be given [15,16]. We proposed that people’s impressions of a patient will be affected by the robot’s behavior. Using text vignettes, we experimentally manipulated the robot’s approach to care: *patient-centered* (focused on the needs and choices of the patient with regard to a treatment plan) or *task-centered* (focusing on how faithfully the treatment plan is being adhered to). We then investigated how the robot’s approach influences: (a) people’s perception of EI in the robot itself, (b) people’s trust in the robot, (c) people’s potential acceptance of the robot for the management of their own health, and finally (d) people’s impressions of the patient. In step 2 of the study, we extended our investigation of social robots’ influence on human-human relationships to other contexts: dieting, learning, and job training.

### Human-Robot Interaction for Health Care Scenarios

Most studies of clinical applications of robots have focused on health outcomes for the patient (see [1]) or on robot acceptance by patients (see [17]). These studies are mainly concerned with the interaction between the patient and the robot, and only very few studies have investigated the effects the robot has on the patient’s interactions with other people.

Several studies have looked at the social effects of PARO, a robot with the appearance of a baby seal that is responsive to touch, sound, temperature, and posture. By serving as the focus of the interaction between people (participants in these studies often interact in pairs or small groups with the robot), PARO was shown to have positive effects on social life such as increasing the density of social networks in a care home for the elderly [8], increasing social activity [18], and increasing social engagement of elderly nursing home residents with varying levels of dementia [19]. PARO was also shown to increase the reported quality of the interaction among people from a nonclinical population [20]. A couple of studies with robots used in therapy for children with autism have also been shown to have effects on the interaction between the child and others. In 1 study, the robot played a mediator role between the therapist and the child by allowing the child to express positive emotion in playing with the therapist [21]. In another study, the robot successfully served as a focus of free play between 2 children [22].

In nonclinical contexts, the social mediation effect of robots has been studied, among others, for the following purposes: conflict resolution with children [23] or adults [24,25], active listening [26], teaching EI through interactive storytelling [27], and enhancing cooperation [28]. Although these results represent a promising start, more research is needed to better understand both the ways in which robots can support social interactions among people and how they might inadvertently influence human-human interactions in perhaps undesirable ways. In clinical contexts, different aspects of human-human interactions, for example, how patients are perceived by others, can have important implications for health care.

### How the Impression of Patient Psychological Attributes Affects Care Decisions

A number of studies have documented the effects of a patient’s affect or perceived personality traits on care decisions; in short, doctors appear willing to prescribe more care for more positive or likable patients. Despite only ranking emotional state as an important consideration in decisions about Intensive Care Unit admission 6% of the time, doctors were almost 3 times more likely to recommend admission of a hypothetical patient if given a vignette that described the patient as upbeat and courageous rather than sad and discouraged [15]. Similarly, another set of doctors recommended more follow-up visits and calls for simulated likable and competent patients and more staff time spent educating simulated likable patients [16].

Other research has indicated that doctors have more positive feelings toward patients who appear happy [29,30] or toward those who express both positive and negative affect over the course of a visit [31]. On the contrary, some primary care physicians report that their challenging patients often become favorites over time and that favorite patients likely benefit from the extra effort that physicians feel inclined to spend on these patients’ care [32].

Doctors are not the only ones who are influenced in their behavior toward patients by the patient’s perceived attributes. A study found that participants (who were not specifically medical professionals) tend to dislike patients who appear distraught but are slightly more willing to aid patients displaying negative affect than those displaying positive affect, offering the least aid to those who show little affect [33].

Given how consequential people’s perceptions of patient psychological attributes are to the patients receiving optimal care, roboticists should be mindful of how they design robots so as not to negatively affect relationships between patients and health care providers or other people. This could also be seen as an opportunity for health care robotics: Social robots could be used to enhance the perception of positive attributes and promote good relationships among patients and doctors, caregivers, or others.

### Patient-Centered Care and Emotional Intelligence as Guidelines for Robot Design

How robots influence relationships in the context of health care, whether in a desirable or undesirable way, will of course depend on their design. More specifically, it will depend on what approach the robot will take for providing care to the patient, and what social capabilities the robot will have.

Patient-centered care [34] is an influential approach to health care that has formulated desirable features for the relationship of the patient with others. This approach emphasizes respect for the patient’s preferences, information, education and communication with the patient, coordination of care, emotional support for the patient, physical comfort, involvement of family, continuity and transition, and access to care [35]. It has been proposed that success in providing patient-centered care may depend on social capabilities such as EI [36].

EI has been linked to positive social relationships in multiple contexts, including health care. EI comprises abilities such as perceiving, understanding, and managing one’s own emotions and the emotions of others. EI has been linked to benefits for both the person possessing these abilities, such as enhanced job performance and stress management [13,37] and better educational outcomes [38], and also for the social group that one is embedded in: EI has been associated with improved teamwork and conflict resolution [39], higher leadership ratings [40], and successful social interactions [41].

In the health care context, EI in medicine has been linked to positive doctor-patient relationships, increased empathy, better teamwork and communication skills, stress management, and organizational commitment and leadership [14]. EI concepts are also central to nursing practice, with implications for nursing students’ learning, ethical decision making, critical thinking, evidence, and knowledge use in practice (for a review see [42]). We thus proposed that EI is needed in social robots that are to operate in the health care setting. To our knowledge, only a couple of studies have so far investigated EI in robots [43,44] and have found that people do expect and detect differences in EI in robot agents and that these differences influence how much robots are trusted [43]. It is thus important to understand people’s perceptions of robots’ EI in the context of patient care.

### Social Robots and Human-Human Relationships Beyond the Health Care Context

Patient care, however, is not the only context in which robots’ influence on relationships between people can have an impact on health and well-being. In the domain of public health, robots could be meaningfully used to mitigate problems such as epidemic obesity and to enhance the relationship between clients and dieticians or weight loss support groups. In the field of education, robots could help promote individualized learning plans and student-centered approaches as well as enhance relationships between students and teachers. In the industry, robots could optimize mentor-trainee interactions and assist in retraining people for new jobs, which has been increasingly needed as technological advances are reshaping the labor market. We have focused the first step of this study on how robots affect relationships in the health care setting, and then we broadened the scope of our inquiry in the second step of the study to other contexts that are more indirectly linked to people’s health and well-being.

### Aims of the Current Study

The aims of this study were to investigate the effects of robot behavior in the health care context and to probe whether similar effects extend to other contexts relevant for well-being and quality of life. In the first step of the study, we investigated whether the robot behavior, patient-centered or task-centered, has an impact on (a) how the robot is perceived, in terms of EI, trustworthiness, and acceptability and (b) the impression that people form of the patient assisted by the robot. We hypothesized that (1) a robot that acts in a patient-centered way will be perceived as having higher EI, (2) inspired by the findings of [43], a robot that acts in a patient-centered way will be trusted more, and (3) accepted more, and we also hypothesized that (4) by acting in a patient-centered way and respecting the patient’s agency, the robot will cause others to think more highly of the patient.

In the second step of the study we further investigated the extent to which the effects observed in the health care context can be replicated and extended to other contexts relevant for people’s health and well-being, contexts in which social robots can provide assistance by monitoring, keeping record, and informing a professional about the user’s progress.

## Methods

### Design

The experiment used a between-group design and a text vignette methodology (see the Materials section). For the first step of the study, investigating the effects of robot behavior in the health care context, we conducted a series of one-way analyses of variance (ANOVA). Our dependent variables were perceptions of robot EI, trust in the robot, robot acceptance, and impression of the patient cared for by the robot (see the Measures section). Our main independent variable was the condition based on which the vignette described the robot’s behavior as being either patient-centered or task-centered (see the Materials section for further details). We also verified for effects of gender as an independent variable and age as a covariate in a series of analyses covariance (ANCOVA).

For the second step of the study, investigating the effects of robot behavior in other contexts relevant for well-being, we used a 2 × 3 between-group experimental design. In addition to the 2 conditions (person-centered or task-centered robot behavior), we also designed vignettes that varied in context (weight loss or learning or job training), which we used as an additional independent variable. As for the health care context, scores from our 4 different questionnaires were used as dependent variables. Gender and participant age were added as variables in the models in a further step.

Our participants were recruited on the Amazon Mechanical Turk (AMT) platform. This made it possible to reach participants from more diverse ethnic backgrounds and spanning a wider age range than what is typical for in-laboratory studies. To ensure language comprehension and a similar compensation incentive, we only recruited participants based in the United States who were fluent in English.

### Participants

For the first step of the study, in which we investigated the effect of robot behavior in the health care context, a total of 199 participants completed the experiment through the AMT. A total of 11 participants failed to pass our attention checks and were excluded from the analyses. According to standard practice for AMT studies, our attention check consisted of a reading comprehension question about the topic of the vignette meant to assess whether participants read it attentively. The 188 participants with usable data ranged in age from 20 to 72 (mean 34.58, SD 10.04) years, 77 were female, and 2 identified as Other. The ethnic composition of the sample was white or Caucasian (74.5%, n=140), Asian (5.8%, n=11), African American (9%, n=17), Hispanic (6.9%, n=13), and Other (3.7%, n=7). In total, 95 participants saw the person-centered vignette and 93 participants saw the task-centered one.

The second step of the study, in which we investigated the effects of robot behavior in other contexts related to well-being, was completed by 299 participants, out of which 254 passed our attention checks, and thus provided usable data. Participants’ ages ranged from 20 to 71 (mean 36.48, SD 10.54) years, and 111 of the participants were female and 5 of the participants identified as *Other*. The ethnic composition of the sample was as follows: white or Caucasian (79.1%, n=201), Asian (3.1%, n=8), African American (7.8%, n=20), Hispanic (6.7%, n=17), and Other (3.1%, n=8). A total of 93 participants saw the weight loss vignette, 92 saw the learning vignette, and 69 saw the job training vignette. The procedure was identical to that in step 1 but with more vignettes. Each participant saw one vignette only, either in the person-centered or task-centered form.

### Measures

#### Perceptions of Robot Emotional Intelligence

We used a 24-item questionnaire based on a measure developed by Caruso and Salovey [45] and previously used by Fan et al. [43] to measure perceptions of EI in robots. Items referred to emotion perception (eg, “Knows why people feel the way they do”), understanding (eg, “Considerate of others’ feelings”), and management (eg, “Creates positive moods in people”) capacities and participants indicated, on a 5-point Likert scale, how much each statement described the robot in the vignette from *0=not at all* to *4=very much so* (Cronbach alpha=.96). We averaged the scores from all the items for each participant.

#### Trust in the Robot

We adapted a 4-item, 5-point Likert scale measure from a study by Mayer and Davis [46] to match the context of the vignette. An example item is “I would be willing to let this robot have control over my health care management.” The measure was adapted for the second step of the study, for example items would refer to weight loss plan instead of health care management for the dieting vignette.

#### Robot Acceptance

Participants rated 2 statements on a 5-point Likert scale from *0=strongly disagree* to *4=strongly agree* about finding the robot useful (“If I were chronically ill, I would find it useful to have a robot like this to help with my treatment”) and wanting to use a similar robot to the one in the vignette (“If I were chronically ill, I would want to use a robot like this to help with my treatment”), should they find themselves in a similar situation to that of the patient. The measure was adapted to match the other contexts for the second step of the study.

#### Impression of Patient

We developed a measure based on the literature investigating the impressions that health care providers have of patient psychological attributes and how that affects their care decisions [15,16,29,47,48]. A set of relevant patient descriptors were collected: dependable and self-disciplined, disorganized and careless, capable of participating in treatment and adhering to health care recommendations, likable, competent, having a positive attitude, defiant, disruptive, hostile, and honest. Participants rated 10 statements about the patient formulated around the descriptors above (eg, “I feel the patient is disorganized and careless”) on a 5-point Likert scale from “0 = strongly disagree” to “4 = strongly agree” (Cronbach alpha=.81). Items indicating negative psychological attributes were reverse-scored. We averaged the scores from all the items for each participant. For consistency and ease of comparison, the person descriptors used in this measure were not changed for step 2 of the study. Note that even though these items were constructed based on the literature on impressions of patient psychological attributes, they are relevant across contexts.

### Materials

For this study, we used the text vignette methodology to evaluate people’s perceptions, attitudes, and impressions toward assistive robots, their behavior, and the patients they care for. In text vignettes, hypothetical situations are described to which participants are asked to respond. This is a common methodology in psychology and sociology experiments and has been used successfully in human-robot interaction research (eg, [43]) as an initial step of investigating and informing possible robot design choices.

**Figure 1:**
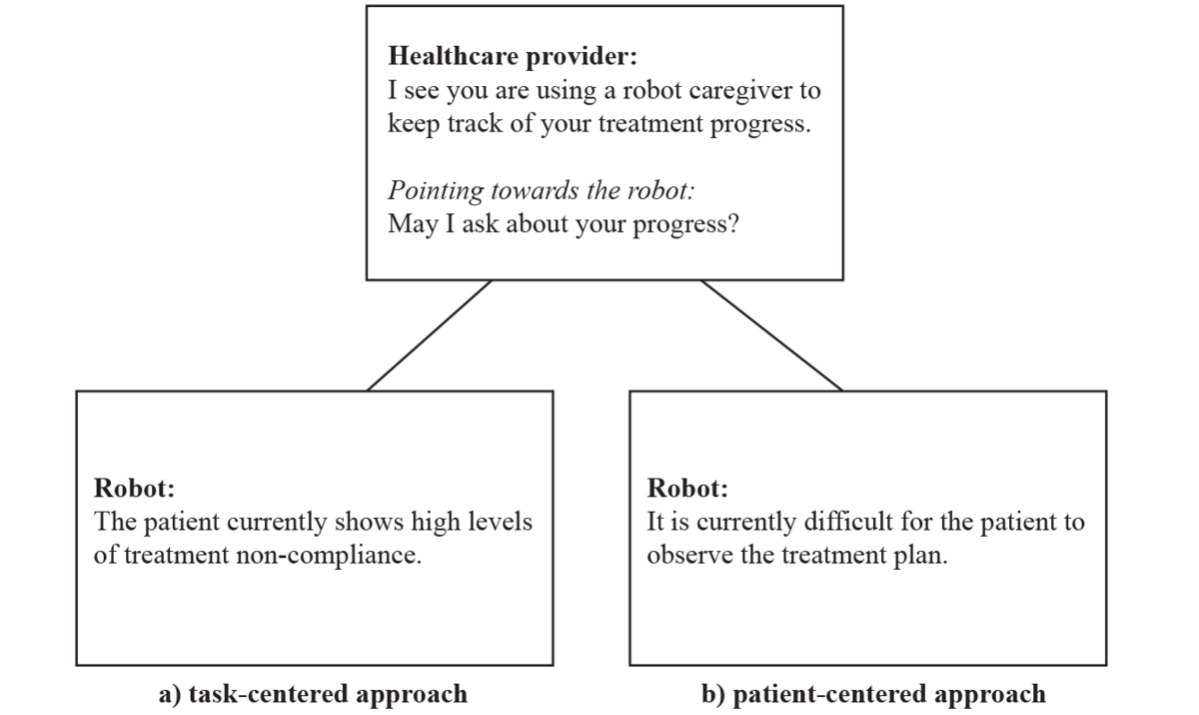
Sample screenshots illustrating the vignette with the 2 conditions: (a) task-centered and (b) patient-centered.

For the first step of the study that focuses on robot behavior in the health care context we used a text vignette featuring 3 characters: a health care provider, a patient, and an assistive robot. The scenario starts with the health care provider noticing that the patient is using an assistive robot for treatment monitoring and asks permission to get a report from the robot about treatment progress, to which the patient responds affirmatively. In one condition, the robot gives a patient-centered report that focuses on the needs and choices of the patient with regard to the treatment plan (patient-centered condition). In the other condition, the robot gives a task-centered report focused on how faithfully the treatment plan was adhered to (task-centered condition; see Figure 1). The treatment plan refers to a medication schedule and a physical exercise routine.

The vignette was presented in a video format. The dialogue was parsed into chunks usually containing one dialogue turn or, in the case of the robot, which speaks for longer into sentences that conveyed one idea. Each chunk was displayed on a blank screen. The text appeared across the screen as if it was being typed (using the typewriter animation effect) with a speed similar to what it would take to say the words out loud. This gave the impression of a dynamic conversation. The speaker was clearly indicated at the beginning of each dialogue turn or chunk of text (eg, “Robot: The patient...”).

The vignette characters were referred to as *health care provider*, *patient*, and *robot*, and no personal characteristics were specified about the human characters (eg, no gender was suggested). In addition, no description of the robot’s capabilities or appearance (eg, whether it was humanoid or not) was provided; rather, this was left to the participants’ imagination. From the vignette, it could be inferred that the robot had the capacity to track treatment progress and produce a spoken report.

For the second step of the study in which we sought to generalize our findings to other contexts, 3 additional vignettes (with 2 conditions each) were used, modeled closely after the vignette in step 1 in terms of (a) structure (ie, the same number of characters and the same conversation progression were kept), (b) theme (ie, the robot monitored progress with regard to a body-oriented task such as dieting and exercising, dancing, and training for a physically active job), (c) conditions (ie, an assistant robot gave a report in a person-centered or task-centered manner), (d) display (ie, the same procedures for chunking and displaying text were used), and (e) amount of information conveyed (ie, no personal characteristics of the humans and no description of the robot were given). The vignettes differed in the context described and the characters involved.

In the first vignette (the dieting vignette), the robot assistant was used for monitoring weight loss progress and the interaction happened between a client, a weight loss coach, and the assistant robot. The weight loss plan involved a meal plan and a physical exercise routine. The second vignette (the learning vignette) featured a robot assistant that was used to keep track of progress in learning how to dance. The interaction took place between a student, a dance instructor, and the assistant robot. The learning goals involved a dance practice schedule and a strength and conditioning routine. Finally, in the third vignette (training vignette), a robot assistant was used for monitoring training progress for volunteer firefighters. The interaction involved a volunteer firefighter, a lieutenant, and the assistant robot. The training goals involved a physical exercise training plan and *Search and Rescue* drill training. Given the diversity of contexts and characters in this second step, we have renamed the *patient-centered condition* from step 1 as the *person-centered condition*, *person* here referring to the weight loss client, the student, or the volunteer firefighter.

### Procedure

Participants accessed the experiment through the AMT. After reading and agreeing to the consent form, participants filled out a demographic questionnaire (age, gender, and ethnicity). They then followed instructions for watching the video. Participants were randomly assigned to one condition: person-centered or task-centered. After watching the video, participants completed the measures in the following order: perception of robot’s EI, trust in the robot, robot acceptance, and impressions of the patient. After concluding the experiment, participants were able to collect their US $1 compensation. All procedures were reviewed and approved by the Institutional Review Board at our university.

## Results

### Perceptions of Robot Emotional Intelligence

#### Step 1: Robot Behavior in Health Care

**Figure 2:**
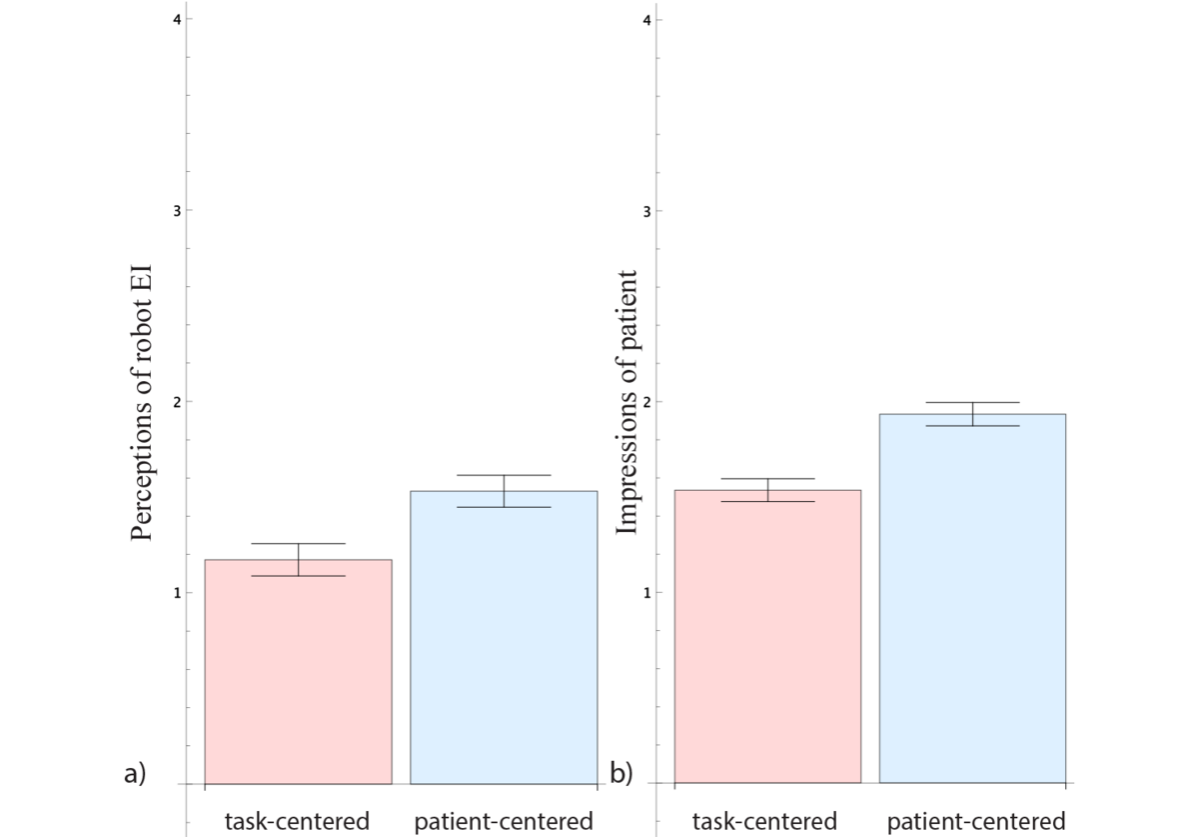
Effect of conditions on (a) perceptions of robot’s emotional intelligence (EI) and (b) impressions of patient (mean and SE).

We began by investigating whether participants perceived a difference in the robot’s EI depending on whether the robot gave a patient-centered or a task-centered report (see the left side of Figure 2 and Table 1).

**Table 1:** Descriptive statistics and ANOVA summaries for the *condition* variable.

Dependent variables	Condition				Statistics		
	Task-centric		Patient-centric				
	N	Mean (SE)	N	Mean (SE)	*F* (df)	*P* value	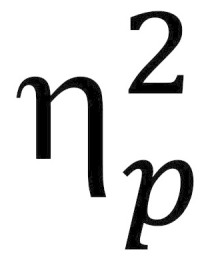
Perceptions of robot emotional intelligence	92	1.17 (0.08)	93	1.53 (0.08)	9.14 (1)	.003	0.05
Trust in the robot	93	1.8 (0.09)	95	1.97 (0.08)	1.84 (1)	.18	0.01
Robot acceptance	92	2.15 (0.13)	95	2.33 (0.12)	1.01 (1)	.32	<0.01
Impression of patient	91	1.53 (0.06)	92	1.93 (0.06)	21.62 (1)	<.001	0.11

We conducted a one-way ANOVA with the *perception of robot EI* as a dependent variable and *condition (patient-centered/task-centered)* as an independent variable. We found a significant effect of *condition*, with the EI of the robot rated significantly higher in the patient-centered condition (*F*_1,183_= 9.14; *P*=.003; and 
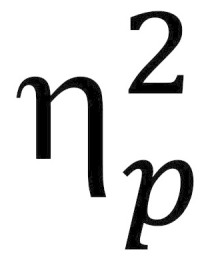
=0.05). We also conducted an ANCOVA, adding the participant’s *gender* (male/female) as an independent variable besides *condition* and the participant’s *age* as a covariate. We again found a significant effect of *condition* (*F*_1,178_=5.99; *P*<.001; and 
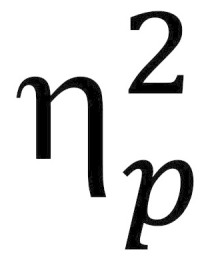
=0.05). No significant effect of participant *gender* or significant interaction between *condition* and *gender* was found. Participant’s *age,* however, had a significant influence on *perceptions of robot EI,* with older participants rating the robot as having lower EI (*F*_1,178_=12.89; *P*<.001; and 
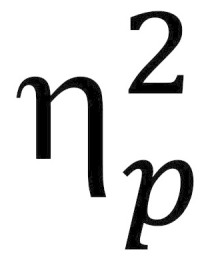
=0.07). This is consistent with the well-known effect of older adults having less favorable opinions of robots in general.

##### Step 2: Robot Behavior in Other Contexts

**Figure 3:**
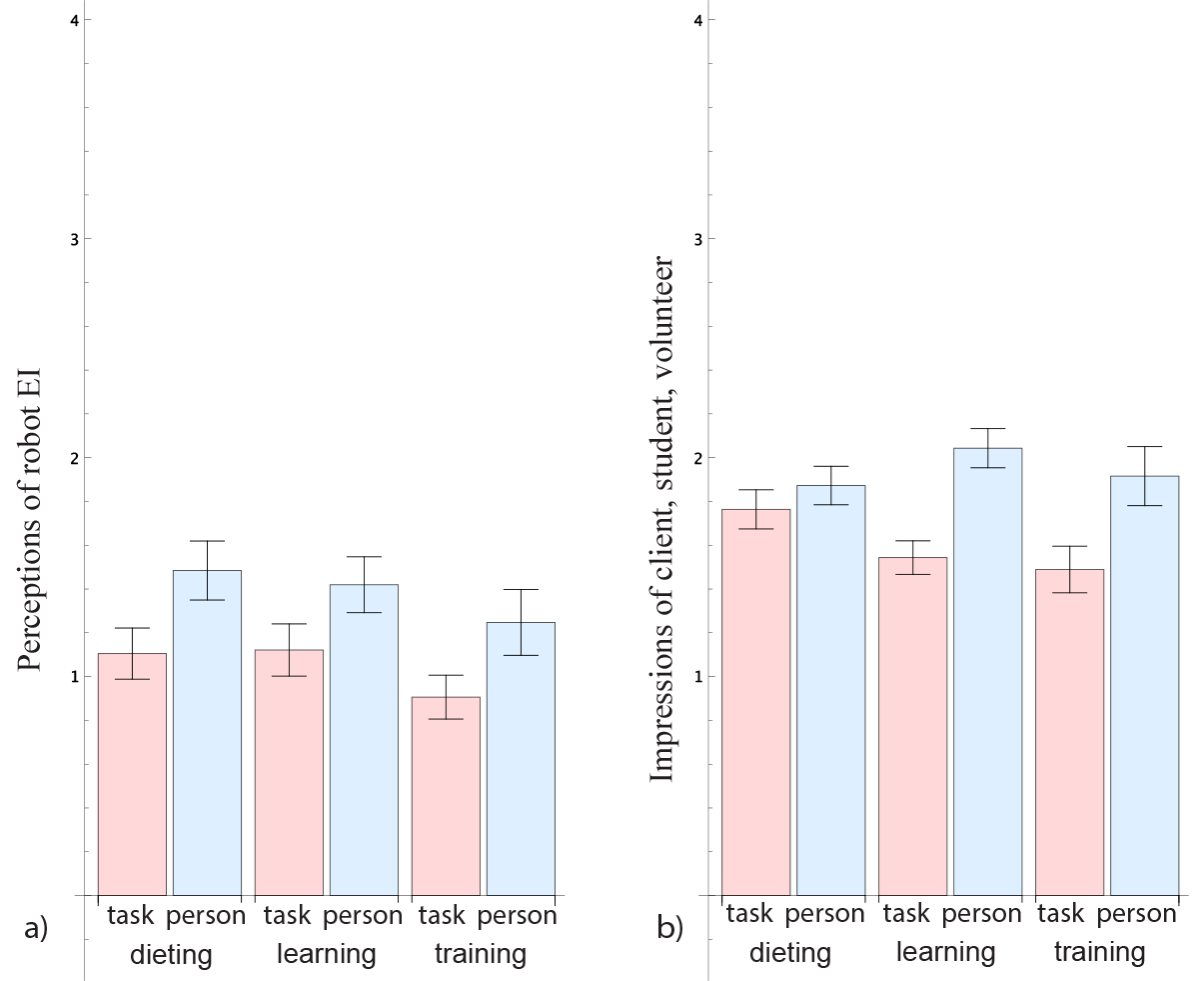
Effects of conditions (task-centered vs person-centered) on (a) perceptions of robot emotional intelligence and (b) impressions of client, student, and volunteer in dieting, learning, and training contexts (mean and SE). Note that for the dieting context, the error bars corresponding to the mean ratings of impression of the client are overlapping between the 2 conditions. Although the same trend is seen as in the other contexts, with higher ratings (ie, more positive impressions) in the person-centered condition, in the case of dieting taken separately, the difference between the conditions is not significant. We list potential reasons in the Discussion section.

Mirroring the first step, we began by investigating whether participants perceived a difference in the robot’s EI based on the condition they were exposed to and the type of context described by the vignette (see left side of Figure 3 and Table 2). We conducted a 2 × 3 ANOVA with *perceptions of robot EI* as the dependent variable and *context (weight loss/learning/job training)* and *condition (person-centered/ task-centered)* as independent variables (see Table 3). We found a significant main effect of *condition*, with participants perceiving higher EI when the robot gave a person-centered report *F*_1,243_=10.71, *P*=.001, and 
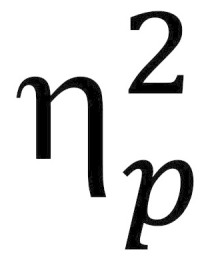
=0.04. The *context* had no significant effect on *perceptions of robot EI*. There was also no significant interaction between the *context* and the *condition*. We also conducted an ANCOVA adding participant *gender* as an independent variable besides *condition* and *context* and participants’ *age* as a covariate. Similar to step 1, we found a significant main effect of *condition* (*F*_1,231_=7.56, *P*<.001, and 
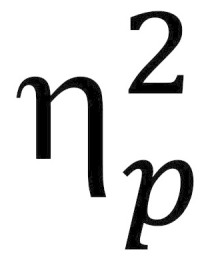
=0.04) and *age* (*F*_1,231_=5.53, *P*=.02, and 
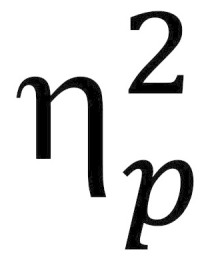
=0.02) but no other significant main effects or interactions.

**Table 2:** Descriptive statistics for the variables *condition* and *context*.

Condition and context			N	Mean (SE)
**Perceptions of robot EI**				
	**Task-centric**			
		Weight loss	48	1.1 (0.12)
		Learning	47	1.12 (0.12)
		Job training	36	0.91 (0.10)
	**Person-centric**			
		Weight loss	43	1.48 (0.13)
		Learning	44	1.42 (0.13)
		Job training	31	1.25 (0.15)
**Trust in the robot**				
	**Task-centric**			
		Weight loss	48	1.97 (0.14)
		Learning	47	1.93 (0.11)
		Job training	37	1.62 (0.18)
	**Person-centric **			
		Weight loss	43	2.17 (0.13)
		Learning	44	2.04 (0.13)
		Job training	32	2.02 (0.17)
**Robot acceptance**				
	**Task-centric**			
		Weight loss	48	1.87 (0.21)
		Learning	47	2.31 (0.17)
		Job training	37	1.69 (0.23)
	**Person-centric**			
		Weight loss	44	2.47 (0.17)
		Learning	45	2.23 (0.19)
		Job training	31	2.27 (0.22)
**Impression of patient**				
	**Task-centric**			
		Weight loss	47	1.76 (0.09)
		Learning	46	1.54 (0.08)
		Job training	37	1.49 (0.11)
	**Person-centric**			
		Weight loss	44	1.87 (0.09)
		Learning	44	2.04 (0.09)
		Job training	32	1.92 (0.13)

**Table 3:** ANOVA summaries for the variables *condition* and *context*.

Variables		Mean squares	*F* (df)	*P* value	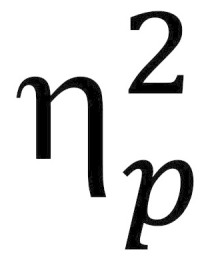
**Perceptions of robot emotional intelligence**					
	Condition	7.00	10.71 (1)	.001	0.04
	Context	1.05	1.60 (2)	.2	0.01
	Condition × context	0.04	0.06 (2)	.94	<0.01
	Residual	0.65	—^a^ (243)	—	—
	Total	0.68	— (248)	—	—
**Trust in the robot**					
	Condition	3.51	4.14 (1)	.04	0.02
	Context	1.22	1.45 (2)	.24	0.01
	Condition × context	0.42	0.50 (2)	.61	<0.01
	Residual	0.85	— (245)	—	—
	Total	0.86	— (250)	—	—
**Robot acceptance**					
	Condition	8.28	5.00 (1)	.03	0.02
	Context	1.64	0.99 (2)	.37	<0.01
	Condition × context	3.21	1.94 (2)	.15	0.01
	Residual	1.65	— (246)	—	—
	Total	1.69	— (251)	—	—
**Impression of person**					
	Condition	7.30	19.30 (1)	<.001	0.07
	Context	0.28	0.74 (2)	.48	<0.01
	Condition × context	0.96	2.53 (2)	.08	0.02
	Residual	0.38	— (244)	—	—
	Total	0.41	— (249)	—	—

^a^Not applicable.

#### Trust in the Robot

##### Step 1: Robot Behavior in Health Care

To investigate whether the robot’s patient-centered or task-centered approach had an influence on how much people trusted the robot, we conducted a one-way ANOVA with *trust in the robot* as the dependent variable and *condition* (*patient-centered/task-centered*) as an independent variable. We found no significant effects of *condition* on *trust in the robot*. We further conducted an ANCOVA with participants’ *gender* as an additional independent variable and the participants’ *age* as a covariate, finding no significant effects of *gender* or *age* on *trust in the robot*.

##### Step 2: Robot Behavior in Other Contexts

We then investigated whether trust in the robot was affected by the person-centered or task-centered conditions or by the vignette context (weight loss/learning/job training). We did this by conducting a 2 × 3 ANOVA with *trust in the robot* as the dependent variable and *condition* and *context* as independent variables. The main effect of *condition (person-centered/task-centered)* was significant *F*_1,245_=4.14, *P*=.04, and 
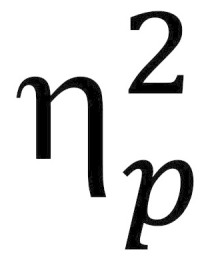
=0.02, with participants in the person-centered condition showing more trust in the robot. An ANCOVA with participants’ *gender* and participants’ *age* added as variables showed no additional main effects. For the ANCOVA, the main effect of condition did not meet 5% significance levels *F*_1,233_=3.52, *P*=.06, and 
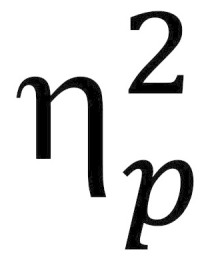
=0.01.

#### Robot Acceptance

##### Step 1: Robot Behavior in Health Care

Next, we explored the potential effect of the robot’s care approach on how helpful and desirable participants would find using such a robot for their own health management. We conducted a one-way ANOVA with *robot acceptance* as the dependent variable and *condition* (*patient-centered/ task-centered*) as an independent variable. We found no significant effects of *condition* on *robot acceptance*. An ANCOVA by adding participant *gender* as an independent variable and participant *age* as a covariate yielded no further significant findings.

#### Step 2: Robot Behavior in Other Contexts

To explore the influence of the 2 conditions as well as that of the vignette context (weight loss/learning/job training) on robot acceptance (participants finding the robot potentially helpful and desirable to use), we conducted another 2 × 3 ANOVA, this time with the *robot acceptance* measure as the dependent variable and *condition* and *context* as independent variables. We again found a main effect of *condition*, with *robot acceptance* being significantly higher for the condition in which the robot provided a person-centered report, *F*_1,246_=5.00, *P*=.03, and 
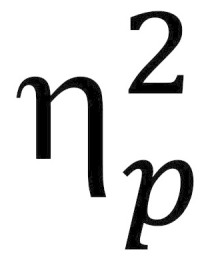
=0.02. As for the other measures, we also conducted an ANCOVA with participants’ *gender* and *age* as additional variables and found no significant main or interaction effects other than that of *condition* *F*_1,234_=4.67, *P*=.03, and 
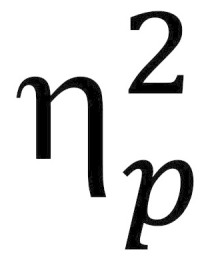
=0.01.

##### Impression of Patient

###### Step 1: Robot Behavior in Health Care

We also examined the effect of the robot’s patient-centered or task-centered behavior on participants’ impressions of the patient’s psychological attributes (see right side of Figure 2) by conducting a one-way ANOVA with the *impression of patient* as a dependent variable and *condition* as an independent variable. We found a significant effect of *condition* on the *impression of patient*, with participants exposed to the patient-centered robot reporting a higher *impression of the patient* score (ie, having a more favorable impression; *F*_1,181_=21.62; *P*<.001; and 
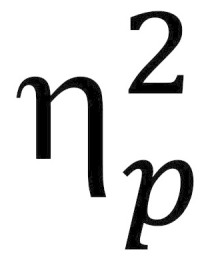
=0.11). We also conducted an ANCOVA with participant *gender* and the interaction between *gender* and *condition* as additional independent variables and participant *age* as a covariate. We again found a significant main effect of *condition*, *F*_1,176_=19.13; *P*<.001; and 
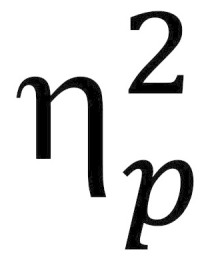
=0.10, and further a significant main effect of *gender*, *F*_1,176_=4.01, *P*=.05, 
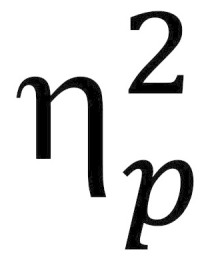
=0.02, with female participants rating their impressions of the patient more positively.

####### Step 2: Robot Behavior in Other Contexts

Finally, we examined the influence of the 2 conditions (person-centered and task-centered robot report) and the context of the vignettes (dieting/learning/job training) on the participants’ impressions of the person (the client, the student, and the volunteer; see Figure 3). We conducted a 2 × 3 ANOVA with the *impression of the person* as the dependent variable and *condition* and *context* as the independent variables. We found a main effect of *condition* *F*_1,244_=19.30, *P*<.001, and 
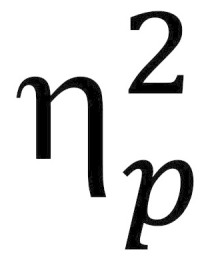
=0.07. Participants in the person-centered condition rated their impression of the client, student, or volunteer as significantly higher (more positive). As before, we proceeded to conducting an ANCOVA, adding participants’ *gender* as an independent variable and participants’ *age* as a covariate. We found no significant main or interaction effects of *gender* or *age* but *condition* emerged again as significant *F*_1,246_=16.16, *P*<.001, and 
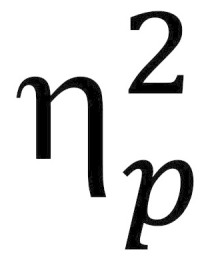
=0.06.

## Discussion

For the health care context, our findings confirm our first hypothesis, namely that robots that exhibit patient-centered behavior are perceived as having higher EI. However, we found no support for our second and third hypotheses: there was no indication that people trusted or accepted the robot less when it behaved in a task-centered way. When Fan et al. [43] found differences in trust based on the robots’ level of EI, their experimental manipulation was much more extreme than the one in this study: They compared a robot that was polite and understanding with a robot that was rude and abrasive.

Also, our results show that the robot’s behavior was able to significantly influence people’s impressions of the patient. It is striking that the robot modeling respect for the patient’s agency positively influenced how people viewed the patient. This is particularly remarkable as the facts reported by the robot were identical: in both conditions, the patient is reported to have failed the same number of times in respecting the medication schedule and following the exercise routine. This suggests new opportunities for using robots to positively influence care for patients.

Beyond health care, we have replicated these results in other contexts relevant to well-being, which shows that these findings generalize to other circumstances. This suggests that social robots’ influence on human-human interactions are not specific to medical treatment adherence but generalize to other categories of interactions. Our findings from step 2 of the study show that robots are perceived as being more emotionally intelligent when they behave in a person-centric way and that their behavior can influence how other people perceive the person they are assisting. In the second step of the study, but not the first, we also found small effects of the robot’s behavior on how trusted or accepted the robot was.

To our knowledge, this is the first investigation that probed the potential influences of assistive robots on how patients are judged by others. By simply varying the way the robot gives a treatment progress report (manipulating the language used, but not the facts conveyed), the robot makes a significant difference to the extent to which the patient is thought of as likable, self-disciplined, competent, and having a positive attitude as opposed to being hostile, disruptive, and disorganized. People take cues from the robot, and when the robot uses a patient-centered language that indicates respect for the patient’s agency, as opposed to a task-centered language that focuses on the treatment benchmarks, people form a more positive impression of the patient.

It is remarkable that robots are able to have this effect given that their language output is scripted and certainly does not come with the emotional connotations that a person’s choice of language would have. A robot’s behavior is not connected to beliefs and attitudes in the same way a human’s behavior is, yet our findings suggest that we inadvertently let our perceptions and impressions of others be guided by the robot’s actions. This can be seen both as having cautionary implications for the field of human-robot interaction but also as an opportunity for the field: if robots can have an influence on how we think of others, then perhaps they can be used for improving relationships in the health care setting and beyond.

Although these results suggest promising possibilities, much more research is needed to understand how assistive social robots can be optimally embedded in social interactions in the health care context. For starters, several gaps and limitations of this study need to be addressed by further research. Because the study is based on hypothetical vignettes, caution is needed in generalizing these findings to real-world interactions. Also, participants in our experiments were laypeople with no connection to the patient. It is not clear whether health care providers or people invested in the patient’s health will be equally susceptible to influence from robots. In addition, participants themselves might have had different amounts of experience with giving care and support to sick individuals, and that might influence how susceptible they are to being influenced by the robot. Also, their own health status might have a bearing on that. Someone struggling with their own health and treatment might be more sympathetic to another person’s need for agency over their own treatment and that might modulate the robot’s influence. It is important to note that variations in cultural norms regarding the care of the sick people, beliefs about how much privacy, agency, and responsibility the patient should have versus the caregivers, will likely influence how people perceive the robot’s behavior as well. The potential interaction of these factors with the effects of the robot behavior remains to be determined through further research.

Another limitation of our study is that the impressions of the patient were formed in the absence of any other information about the patient. In real-life scenarios, even for very short, first-time interactions, people give off an abundance of signals about who they are (age, gender, ethnic group, and social class) and research has shown that humans are able to form impressions extraordinarily fast [49]. It is therefore likely that the robot’s effect on people’s impression might be greatly modulated by the information conveyed by the patient herself. For example, people might hold different beliefs to start with in terms of how much agency someone should have over their own treatment based on whether they are an older adult versus a young adult versus a child. In our vignettes, people’s characteristics were left completely to the participant’s imagination, and some might have formed very vivid images of the patient whereas others less so.

Similarly, the robot characteristics were left to the imagination of the participants, which might have contributed to how the robot was perceived. The contributions of various characteristics to the robot perception are likely mixed. For example, the findings of Fan et al [43] suggest that some characteristics are very resistant to changes in the perception of EI in robots: the robot’s voice and gestures did not have an influence on people’s ratings of the robot’s EI, the authors finding no differences between video, audio, or text vignettes. It seems that what the robot says is the single most important factor in how emotionally intelligent the robot is perceived. However, another study [44] found that the robot assigned gender (indicated through name), in addition to what the robot said influenced how emotionally intelligent the robot was perceived to be.

Finally, although we found that the robot’s influence on others’ impression of the person the robot is assisting is generalizable to a variety of contexts, the magnitude of the effect might vary from context to context. Figure 3 suggests that people are not equally susceptible to having their impressions influenced by the robot’s behavior in the dieting context. For dieting, the task-centered behavior of the robot does not negatively affect people’s impression of the person trying to lose weight. Perhaps people have more experience with dieting and how difficult it can be to adhere to a dieting plan, and thus feel more empathy toward the weight loss coaching client. Another possibility is that people already feel that individuals should have agency and choices over their weight, much more so than over their medical treatment, studies, or job performance. Further research is needed to determine the context-related factors that influence how people form their opinions of others.

### Conclusions

This is the first study, to our knowledge, to investigate the influence of robots’ behavior on the impressions that people form about the person the robot is assisting. We found that people perceived the robot as having higher EI when it was behaving in a person-centric way and in some contexts as being more trustworthy and having a higher acceptance rate. Most importantly, robots were able to influence people’s impressions of patients, coaching clients, students, and volunteers by modeling behavior that was respectful of these people’s agency and choices with regard to medical treatment, weight loss plans, learning, and job training. The most immediate implications for human-robot interaction research are that (1) it is important to be aware of the social assistive robots’ influence on the relationships between the people assisted and others and (2) the ability to positively impact human relationships opens up new exciting opportunities for the use of robots in pursuing health and well-being.

## Abbreviations

AMT: Amazon Mechanical Turk
EI: emotional intelligence
